# Supplementary figures and images for: Improving pediatric care in Uganda with a digital platform and quality improvement initiative: A retrospective review of Smart Triage + QI
Source: PLoS One. 2025 Aug 7;20(8):e0329369. doi: 10.1371/journal.pone.0329369 (PMC12331088; doi:10.1371/journal.pone.0329369)

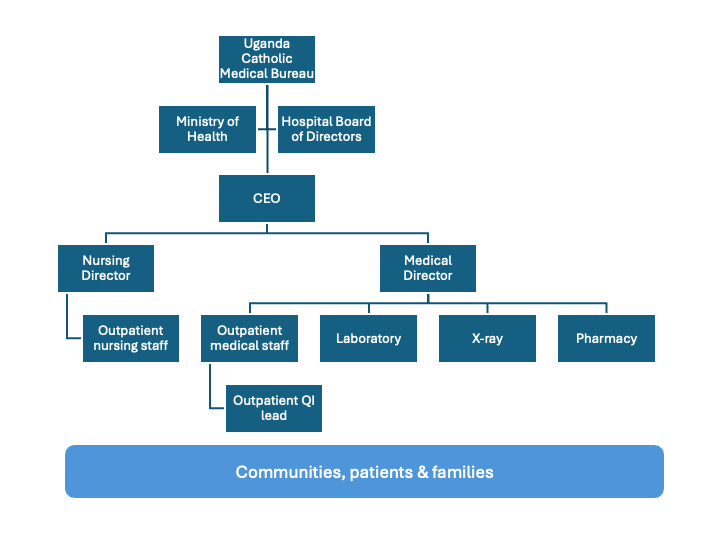

Supplement: S1 Fig — (TIFF) [file pone.0329369.s001.tiff]
