# Supplementary material for: Improving pediatric care in Uganda with a digital platform and quality improvement initiative: A retrospective review of Smart Triage + QI
Source: PLoS One. 2025 Aug 7;20(8):e0329369. doi: 10.1371/journal.pone.0329369 (PMC12331088; doi:10.1371/journal.pone.0329369)
Supplement: Attachment — Submitted filename: ResponseToReviewers.docx [file pone.0329369.s004.docx]

25^th^ June 2025

Re: **Improving pediatric care in Uganda with a digital platform and quality improvement initiative: a retrospective review of Smart Triage + QI**

We want to thank the reviewer for their comments. We appreciate your efforts to improve the manuscript. We address each of their points below. Each point is presented on a light grey background; our response is underneath, with manuscript text in italics and any additions to the text in bold.

We have made two additional modifications to the manuscript.

We hope that we have addressed the reviewers’ questions adequately and that you find our proposed revisions to the manuscript acceptable.

Sincerely,

**Dr J Mark Ansermino**

Department of Anesthesiology, Pharmacology & Therapeutics | Institute for Global Health

The University of British Columbia | BC Children's Hospital

938 W 28th Ave | Vancouver BC | V5Z 4H4 Canada, Phone 604 997 7753, [mansermino@bcchr.ca](mailto:mansermino@bcchr.ca)

# Reviewer #2

Reviewer #2: The new version of the manuscript clarifies almost all the doubts we had raised.
However, there are a couple of observations that we suggest to the authors that could further improve their manuscript.

• The authors state that they cannot analyse specific mortality rates for patient characteristics, as they do not have a data source that allows linkage with the patients included in the study. However, in response to a question about a statement at the beginning of the paragraph Evaluatyon and analysis
'Lines 220-222: "HICH's primary outcomes were the triaging rates within the OPD, the median time from triage to initiation of antimicrobials (minutes), and the mortality rates of admitted patients at HICH”. Why was the evaluation of mortality rates not stated early on as one of the objectives of the study?
the authors answer: “The individual studies' numbers were too small to expect a significant decrease in mortality, and the studies were not powered to demonstrate a reduction in mortality".
It is therefore unclear whether mortality among the subjects studied was not calculated due to low numbers of observed events or whether it was not among the objectives of the study anyway, since the study was not sized to assess it. It would be interesting to understand this, since mortality rates of admitted patients at HICH is counted among the primary outcomes of the study.

•

Thank you for your insightful review of the results! As previously indicated, two prospective studies were conducted, followed by a subsequent retrospective quality improvement project, which is reported in this manuscript. We do have mortality data in the two prospective studies; however, this is limited to the subjects recruited during the study periods. The study team does not have data on the patients who were not recruited, those seen between the two studies and after the second study. The mortality data we report in this manuscript is from the facility mortality reports. This is limited to in hospital mortlity data.

We have clarified this in the manuscript.

Line 239-242: HICH’s primary outcomes were the triaging rates within the OPD, the median time from triage to initiation of antimicrobials (minutes), and the mortality rates of admitted patients **from the monthly facility mortality reports at HICH (mortality data for triaged patients was only determined during the study periods).**

Line 266-269: The overall trajectories of change of OPD attendance, triaging rates, illness acuity (independent ED-PEWS urgency categories), time-to-antimicrobials, and mortality rates **from the facility monthly statistics of all patients** were assessed using simple linear regression with time as the explanatory variable.

The authors responded to the observation that “The OPD triage rate at the end of the observation period is broadly similar to that in 2018, before the implementation of the Smart Triage platform” (about 90%) that “The reason for the high triage rate at the start of the observation is that the triage was supported by the study team during the baseline period”. They modified the text accordingly. Would it be possible to have an estimate of the triage rate before the start of this support before the baseline period?.

In this facility, as in many facilities in LMICs, there was no formal triage process in place before the feasibility study in 2018. While the WHO ETAT guidelines were used, this does not dictate a formal process of seeing patients in a specific order. The nurse who would perform the initial observation may determine that the patient was critically ill, but this information was not recorded.
